# New role for a CEP peptide and its receptor: complex control of lateral roots

**DOI:** 10.1093/jxb/erw306

**Published:** 2016-08-13

**Authors:** Michael Taleski, Nijat Imin, Michael A. Djordjevic

**Affiliations:** Division of Plant Sciences, Research School of Biology, College of Medicine, Biology and Environment, The Australian National University, Canberra ACT 0200, Australia

**Keywords:** *In vivo* peptide identification, local signalling, long-distance signalling, optimising nutrient uptake, peptide hormones, root system architecture.

**Optimized root system deployment should enable more-efficient nutrient acquisition and increased crop yields. C-TERMINALLY ENCODED PEPTIDE (CEP) hormones and their receptors, which regulate root growth, could be important in research with this aim. Roberts *et al.* (pages 4889–4899 in this issue) suggest that the full extent of CEP function and signalling is highly complex, and we emerge with a picture of CEPs and their known receptors being involved in long-distance and possibly more local regulatory networks.**

The initiation, formation and elongation of lateral roots are key determinants of root system architecture which is, in turn, critical to effective nutrient and water acquisition and plant growth. These periodic, postembryonic organs arise along the main root from the asymmetric cell divisions of a specific subpopulation of pericycle cells in Arabidopsis. How the root system deploys them and why different species display distinct root system architectures has been an ongoing area of intense study. Previous work has established that lateral root development and nutrient uptake in a plant in a spatially heterogeneous soil environment is controlled by local and systemic responses (Zhang *et al.*, 1999; Ruffel *et al.*, 2011; Mounier *et al.*, 2014). Now, Roberts *et al.* (2016) propose a local role for Arabidopsis CEP5 and its receptor in regulating lateral root initiation.

CEPs are secreted, 15-amino-acid peptide hormones that are post-translationally modified (Ohyama *et al.*, 2008; Mohd-Radzman *et al.*, 2015). They control root growth and development by interacting with membrane-bound receptors on target cells (Tabata *et al.*, 2014; Mohd-Radzman *et al.*, 2016), and are encoded by a widely distributed multigene family in seed plants that is absent in early plant lineages (Roberts *et al.*, 2013; Delay *et al.*, 2013; Ogilvie *et al.*, 2014). AtCEP1 was first described as a negative root growth regulator (Ohyama *et al.*, 2008). Further work showed that *CEP* genes are induced under nutrient or abiotic stress conditions, particularly nitrogen (N) limitation (Delay *et al.*, 2013; Imin *et al.*, 2013), and this is strongly supported by several publically available transcriptomic datasets.

Tabata *et al.* (2014) identified two CEP receptors (CEPRs) that bind CEP peptides [XYLEM INTERMIXED WITH PHLOEM 1 (XIP1)/CEPR1 and CEPR2], and provided evidence for long-distance CEP movement being implicated in N-demand signalling. Here, CEPs synthesized in roots under N-limitation travel in the xylem stream to the shoot to bind CEPRs. This produces an unknown return signal that up-regulates important high-affinity nitrate transporter genes in roots under more favourable N conditions. This enables roots in a more favourable N-microenvironment to profit from the better local conditions, perhaps to compensate for roots on the same plant in less favourable local microenvironments. The systemic control of nitrate transporter transcription appears to act through XIP1/CEPR1. The systemic up-regulation of nitrate transporter transcription by long-distance CEP signalling was specifically demonstrated by Tabata *et al.* (2014); however, a systemic enhancement of lateral root formation by the same mechanism was not reported. Whether or not control of lateral root development fits into this N-demand model has not been determined. In addition, no phenotype was attributed to CEPR2 and no role for root-located CEPR1 and CEPR2 was reported.

## New insight into CEP signalling

To take full advantage of CEP signalling to optimize root system architecture, a more comprehensive understanding of the environmental and developmental context in which CEPs and their receptors operate is required. One view is that the function and regulation of CEPs is a part of the plant’s response to low N. CEP expression, however, is tightly linked with lateral root primordia under N-replete conditions (Roberts *et al.*, 2013; Tabata *et al.*, 2014). With auxin established as integral to lateral root development (Lavenus *et al.*, 2013), it is not surprising that control of lateral roots by CEPs may involve interactions with this phytohormone. Indeed, Roberts *et al.* (2016) show that auxin negatively regulates *CEP5* expression. They propose that an auxin minimum in phloem pole pericycle cells up-regulates CEP5 expression near to, but not at, lateral root initiation sites. An auxin maximum occurs at the actual LRI site. It will be interesting to see the extent to which the low-N-centric view of CEP function can be unified with auxin interactions, as lateral root control under low-N involves auxin signalling (Zhang *et al.*, 1999; Mounier *et al.*, 2014).

## How do CEPs act through XIP1/CEPR1?

Tabata *et al.* (2014) and Roberts *et al.* (2016) provide different perspectives into CEP-controlled processes through XIP1/CEPR1 (Box 1). Whilst Tabata *et al.* (2014) show that several CEPs are low-N-dependent root-to-shoot signals, it is conceivable that CEP5 may act locally through XIP1/CEPR1 in control of lateral root initiation, since Roberts *et al.* (2016) show they are co-localized in the root at phloem pole pericycle cells. Precedence for local control of lateral roots by CEP signalling occurs in *Medicago truncatula*, where lateral root number is inhibited in *MtCEP1* overexpressing root cultures that lack shoots (Mohd-Radzman *et al.*, 2015). Additionally, COMPACT ROOT ARCHITECTURE 2 (CRA2), a XIP1/CEPR1 homologue and likely *M. truncatula* CEP receptor (Mohd-Radzman *et al.*, 2016), was shown to locally control lateral root density using grafting (Huault *et al.*, 2014).

**Box 1. Models for CEP signalling through the receptor XIP1/CEPR1**

Model 1: Tabata *et al.* (2014) suggest that CEPs produced under local low N act as long-distance signals through shoot-located XIP1/CEPR1 to up-regulate high-affinity nitrate transporter genes in roots under local high N. Model 2: Roberts *et al.* (2016) suggest CEP5, expressed at an auxin minimum, may act locally to inhibit lateral root initiation. This may be dependent on root-located XIP1/CEPR1.

**Figure:**
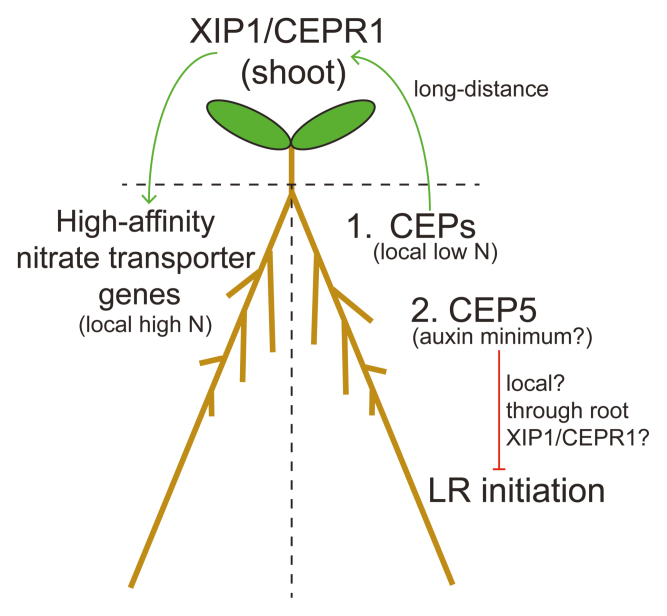

Roberts *et al.* (2016) also open up the possibility that some CEPs may act as antagonists since the *AtCEP5* knockdown and *xip1-1* mutants have opposite lateral root initiation phenotypes. Indeed, antagonistic relationships exist in other peptide hormone systems. The STOMAGEN and EPIDERMAL PATTERNING FACTOR 2 peptides bind ERECTA and its co-receptor, TOO MANY MOUTHS, to positively and negatively regulate stomatal development, respectively (Lee *et al.*, 2015).

## Identifying CEP species *in vivo*

It is important to identify and validate *in vivo* CEP peptide hormone structures and determine the biological relevance of their post-translational modifications. Both Roberts *et al.* (2016) and Tabata *et al.* (2014) used mass spectrometry to identify CEP species *in vivo*. Tabata *et al.* (2014) identified multiple, post-translationally modified, CEP species in xylem sap from low-N-grown plants, including CEP5, and presented MS/MS spectra to verify their findings. These results are consistent with other studies (Ohyama *et al.* 2008; Mohd-Radzman *et al.*, 2015). Roberts *et al.* (2016) putatively identified the same CEP5 species as in Tabata *et al.* (2014), except that they used extracted root material. This is the first detection of a putative CEP peptide directly from extracted plant material; however, this species was detected at the limits of detection using only a single internal mass tag. Currently, validation of peptide hormones *in vivo* remains a difficult technique restricted to a few laboratories. Therefore, more broadly adaptable methods and approaches are required to enable this technique to be more widely used so that a full diversity of CEP peptide hormones, and indeed other peptide species, can be determined.

## Where next?

Whilst the results of Roberts *et al.* (2016) and Tabata *et al.* (2014) may appear inconsistent, this is probably because the full story is yet to be uncovered. The two studies use mutants in different Arabidopsis backgrounds (Col-0 and Nössen, respectively), and focus on control of different processes (lateral root initiation and systemic control of nitrate transporters, respectively). To clarify roles for CEP signalling through XIP1/CEPR1, further work is required examining these processes in both backgrounds. A lack of *Arabidopsis XIP1/CEPR1* knockout mutant alleles is not ideal for drawing firm conclusions about its roles, especially since *xip1-1* is a missense mutation and *cepr1-1* is an insertion knockout. In contrast, *CRA2* in *M. truncatula* has more than ten knockout mutant alleles with consistent, highly branched root phenotypes. In *cra2* mutants, lateral roots emerge rapidly and independently of growth conditions (Huault *et al.* 2014). Characterizing more receptor and CEP gene mutants in *Arabidopsis* could help rectify any uncertainty.

Roberts *et al.* (2016) reveal new insights into CEP function and signalling and suggest that it is likely to be both complex and context dependent, and involve local relays. Gaining a more complete picture of CEP signalling in different systems is important for understanding how plants can regulate growth and development and adapt to changing environments. It is also necessary for CEP signalling to be exploited in biotechnological applications to optimize root systems.
